# Three-Month Safety and Efficacy Outcomes for the Smaller-Incision New-Generation Implantable Miniature Telescope (SING IMT™)

**DOI:** 10.3390/jcm12020518

**Published:** 2023-01-08

**Authors:** Mario Damiano Toro, Faustino Vidal-Aroca, Marina Montemagni, Claudio Xompero, Gaetano Fioretto, Ciro Costagliola

**Affiliations:** 1 Eye Clinic, Department of Public Health, University of Naples Federico II, 80131 Naples, Italy; 2 Chair and Department of General and Pediatric Ophthalmology, Medical University of Lublin, 20079 Lublin, Poland; 3 Department of Scientific Affairs, Medevise Consulting, 16 avenue de l’Europe, 67300 Schiltigheim, France; 4 Eye Clinic, Department of Neurosciences, Reproductive and Dentistry Sciences, University of Naples Federico II, 80131 Naples, Italy

**Keywords:** small-incision new-generation implantable miniature telescope, end-stage age-related macular degeneration, visual prosthesis, low vision, geographic atrophy, visual impairment

## Abstract

The smaller-incision new-generation implantable miniature telescope (SING IMT™) is the second generation of the IMT™, a telescope prosthesis that is indicated for monocular implantation in patients with stable vision impairment caused by bilateral central scotomas associated with end-stage Age-related macular degeneration (AMD). This non-comparative retrospective study is the first and largest single-surgeon case series to evaluate the short-term (3 months) safety and efficacy of the device in patients with disciform scars or geographic atrophy at baseline. The main outcome measures included best-corrected distance and near visual acuity (CDVA and CDNVA, respectively), endothelial cell density (ECD) loss, and the incidence of complications. At postoperative month 3 in the study eyes, mean CDVA and CDNVA improved by +14.9 ± 7.1 letters and +7.7 ± 3.2 Jaeger levels, respectively. Importantly, 70.83% of patients gained ≥ 2 lines, 58.33% ≥ 3 lines, and 25.00% ≥ 4 lines of CDVA. From baseline, ECD loss in the study eyes was 10.4 ± 13.3% at 3 months, however, ECD was comparable between the study and fellow eyes at all time points. The most common complication was corneal edema. In all, these short-term outcomes suggest that the SING IMT™ delivers lower ECD loss than the first-generation IMT ™, but similar visual outcomes and safety.

## 1. Introduction

Age-related macular degeneration (AMD) is a neurodegenerative disease that preferentially affects the central retina, i.e., the macula, which accounts for almost 10% of the entire visual field and the majority of vision under bright light, i.e., photopic vision [1,2]. Although a multifactorial disease, it is characterized by progressive central visual impairment resulting from the degeneration of the photoreceptor–retinal pigment epithelium complex. 

It is the leading cause of visual disability in individuals aged 55 years and above in the developed world, accounting for 6–9% of legal blindness globally [3]. Approximately 11 million individuals in the United States (US) alone and 170 million individuals globally have some form of AMD, broadly categorized into early, intermediate, and advanced AMD [1]. Due to an increasingly aging population, this prevalence is expected to increase to 22 million in the US by 2050 and 288 million globally by 2040 [1,3]. 

The advanced stages of AMD include exudative or ‘wet’ AMD, characterized by choroidal neovascularization (CNV), and geographic atrophy (GA) resulting from non-exudative or ‘dry’ AMD. Only 10–15% of patients with intermediate AMD progress to wet AMD, with the remaining 85–90% of patients developing GA or a disciform scar associated with CNV [4]. Unlike the conversion to wet AMD, which can occur rapidly, GA lesion progression can be slow and variable [5]. These patients can have relatively stable vision and insignificant lesion growth for years before experiencing rapid and profound central vision loss. Moreover, unilateral GA can develop into bilateral GA within a median of 7 years [6]. Patients with GA experience profound central and peripheral vision loss compared to their age-matched counterparts, and this affects a range of daily near, intermediate, and distance activities [7,8]. Unsurprisingly, vision impairment caused by GA results in increased functional disability and loss of independence which, in turn, increases the risk of clinical depression and anxiety in these patients and an overall poorer quality of life [9]. 

There has been considerable and ongoing success in the development of therapeutics for wet AMD, ranging from laser procedures to anti-neovascular agents, which provide significant improvements in vision for a large proportion of patients [2]. Given the complex etiology of dry AMD, while there are currently no treatments available for patients with GA, clinical trials evaluating therapies that may slow the progression of GA are ongoing [10]. However, the only non-surgical options for visual rehabilitation available to patients experiencing central vision loss, due to either form of advanced AMD, include low vision aids such as spectacles, head-mounted or hand-held telescopes, and magnifiers [11,12]. Although these aids are widely available, they are not without limitations. For example, extraocular aids require patients to learn to scan their visual field by physically moving their head or hands rather than using natural eye movements. Not only can this lead to motion sickness due to vestibular–ocular conflict, but patients with comorbidities such as arthritis or neuromuscular disorders may find it challenging to maneuver them [12]. Moreover, patients find these devices bulky, uncomfortable, and unattractive. 

In the past two decades, intraocular, vision-improving devices, such as implantable telescopic devices and intraocular lens implants, have been developed as attractive low vision rehabilitation alternatives. The Galilean implantable miniature telescope (IMT™, Vision Care Ophthalmic Technologies, Saratoga, CA) was first evaluated in the prospective 2-year multicenter IMT-002 pivotal study including 217 patients with bilateral end-stage AMD [13,14,15]. The study demonstrated that 90% of implanted eyes gained 10 or more letters on the Early Treatment Diabetic Retinopathy Study (ETDRS) chart, i.e., a two-line or greater improvement, in best-corrected distance or near visual acuity (CDVA or CDNVA) from baseline up to 60 months, and an overall improvement in patient quality of life [14,15,16]. The IMT™ is currently approved by the US Food and Drug Administration (FDA) and Canadian and European authorities for monocular implantation to improve the vision of patients aged at least 65 years with stable severe to profound vision impairment (CDVA 20/160 to 20/800) caused by bilateral central scotomas associated with end-stage AMD [16]. 

In 2020, the second generation of the implant, termed the smaller-incision new-generation implantable miniature telescope (SING IMT™) was developed and approved for use within the European Union. Like its predecessor, the SING IMT™ has the same magnification, optics diameter, and axial height; however, unlike its predecessor, it has a smaller overall diameter, requires fewer sutures, and has greater postoperative corneal clearance [11], potentially leading to lower rates of endothelial cell density (ECD) loss. Importantly, the rigid poly(methyl methacrylate) (PMMA) haptics have been replaced with flexible silicone haptics that allow the SING IMT™ to be injected through an injector, thereby substantially reducing the corneal incision size from 10–12 mm to 8.0 mm [17]. Taken together, these improvements were intended to make the surgical procedure shorter, safer, and simpler.

This retrospective study is the first and largest single-surgeon case series to describe the short-term (3 months) safety and efficacy outcomes of the SING IMT™ prosthesis in patients with severe to profound bilateral central vision impairment due to advanced AMD. Briefly, the device was demonstrated to be safe, and produced considerably lower levels of ECD loss in comparison to the IMT-002 pivotal study evaluating the IMT™ [14]. Distance and near visual acuity achieved with the SING IMT™ at 3 months were on par with those gained with the IMT™ at 12 months. Taken together, the SING IMT™ represents a significant step forward in the evolution of intraocular low vision aids.

## 2. Materials and Methods

This retrospective single-center clinical study included all consecutive phakic eyes with end-stage AMD treated at the Department of Ophthalmology, University of Naples Federico II, Italy, from February 2022 to June 2022. The study was performed in accordance with the tenets of the Declaration of Helsinki and approved by the Ethics Committee of the University of Naples Federico II (n° EC-19/2022). Written informed consent for the processing of personal data was obtained from all patients. 

### 2.1. Inclusion and Exclusion Criteria

Patients included in the study had to be at least 55 years of age, have stable central visual acuity loss caused by untreatable bilateral end-stage AMD (geographic atrophy, disciform scar, or both), be phakic with a diagnosis of cataract in the study eye, and have good peripheral vision in the fellow eye (i.e., no other retinal diseases). Patients were required to have an anterior chamber depth (ACD) greater than 2.5 mm, endothelial cell density (ECD) greater than 1600 cells/mm^2^, intraocular pressure (IOP) less than 22 mmHg, and bilateral CDVA between 20/80 and 20/800 on the ETDRS visual acuity chart. The study eye had to be able to achieve at least a five-letter improvement on the ETDRS scale using an external telescope simulator (ETS 3.0X-FF; VisionCare Ophthalmic Technologies Ltd., Petah Tikva, Israel) prior to surgery. The SING IMT™ implant was placed in the eye with the potential for greater gain in ETDRS letters, as determined with the ETS. Patients were also screened for their availability during the study and willingness to attend all evaluation, testing, and rehabilitation visits and participate in postoperative vision training with a low vision specialist.

Patients with active choroidal neovascularization (CNV) or those who had undergone treatment for CNV in the preceding 6 months, a history of intraocular or corneal surgery, presence of corneal stromal or endothelial dystrophies (including guttae), myopia greater than 6.0 D, hyperopia greater than 4.0 D, a history of steroid-responsive increases in IOP, preoperative IOP greater than or equal to 22 mmHg while on maximum medication, or uncontrolled glaucoma in the study eye were excluded from the study. The presence of ophthalmic pathology that could compromise peripheral vision in the fellow eye was also exclusionary. Finally, patients with marked cognitive impairment, which could interfere with their ability to understand instructions, follow directions, or undergo proper visual training/rehabilitation with the implant, were also excluded. 

### 2.2. Examination Methods 

Patient demographics and ophthalmic preoperative and postoperative data were derived from the electronic medical records. All patients underwent a thorough ophthalmic examination, including slit lamp biomicroscopy, gonioscopy, dilated fundus examination, and measurements of CDVA, CDNVA, IOP, and corneal ECD. 

Patients were examined at baseline and postoperative day 1, day 15, month 1, and month 3. Best-corrected distance and near visual acuity were measured using the ETDRS chart or Jaeger levels (J), respectively. Intraocular pressure was measured using Goldmann applanation tonometry, and preoperative ACD was assessed with ultrasound biomicroscopy (UBM; ParadigmMedicalIndustries, Salt Lake City, Utah). Ocular imaging was performed using spectral domain optical coherence tomography (SD-OCT; Spectralis, Heidelberg Engineering, Heidelberg, Germany), optical biometry (OA-2000 optical biometer, TOMEY GmbH, Nuremberg, Germany), and endothelial microscopy (Perseus, CSO, Florence, Italy). Preoperative simulation of the intervention was performed using the hand-held ETS.

Patients participated in visual rehabilitation sessions from postoperative week 4 onwards, undergoing at least four rehabilitation sessions, each taking place once every 3 weeks.

### 2.3. Surgical Technique 

All surgeries were performed by the same surgeon (M.D.T). Anesthesia was induced 10 min before surgery by peribulbar block. Following iris dilation, a conjunctival peritomy was performed at the 12 o’clock position. Scleral bleeding was controlled using bipolar coagulation forceps. Per standard cataract extraction procedure, a 2.75 mm sclerocorneal tunnel was built on three levels and positioned approximately 1 mm from the limbus in the upper corneal quadrant, at 12 o’clock. A dispersive ophthalmic viscosurgical device (OVD) was then injected into the anterior chamber. Two side-service 1.2 mm paracentesis incisions were placed at the 5 and 7 o’clock positions and continuous manual circular anterior capsulorrhexis was carried out with a diameter of at least 5.5–6 mm. Cataract hydrodissection, hydrodelamination, and nucleus rotation were then performed before phacoemulsification and thorough cortical cleanup. 

The SING IMT™ implant comes pre-loaded within the Tsert delivery system (including cartridge and injector). Prior to implantation, the anterior chamber and capsular bag were filled with viscoadaptive/cohesive OVD, and a dispersive OVD was used to coat the inner walls of the injector tip. The sclerocorneal tunnel was enlarged up to at least 8 mm, as it is challenging to insert the implant through a smaller cut. During injection, the injector tip was maintained at an approximately 45° angle to the corneolimbal incision. When the injector tip reached the capsulorrhexis plane, the syringe was gently angled to ensure that the angulated edge of the injector tip was beneath the capsulorrhexis plane and completely within the bag. Once the SING IMT™ was correctly implanted with two haptics positioned inferiorly and one haptic positioned superiorly in the capsular bag, three single sclerocorneal sutures (10-0 monofilament nylon) were performed. After the OVD was removed, the incision was sealed with 10-0 nylon sutures, with approximately 1 mm spacing between sutures. 

### 2.4. Postoperative Care 

A standardized postoperative regimen of topical ophthalmic medications was used in this study. One drop of levofloxacin/dexamethasone eyedrops was administered every 6 h over the first 7 days, followed by one drop of dexamethasone eyedrops every 8 h for the next 21 days. Two drops of a non-steroidal anti-inflammatory drug (bromfenac) eyedrop, a mydricatic agent (tropicamide 100 mg/mL), and a cycloplegic agent (phenylephrine 5 mg/mL) were administered.

In the event of corneal edema on postoperative day 1, one drop of a hypertonic eyedrop was administered three times a day for the first 15 days.

### 2.5. Statistical Analysis 

All measured variables and derived parameters were listed individually and, if appropriate, summarized using descriptive statistics. Demographic information included gender, diagnosis, implanted eye, and age in years. Efficacy parameters included CDVA, CDNVA, ACD, ECD, and IOP. Additionally, visual acuity parameters were categorized into loss, no change, and improvement categories based on the number of letters that were gained or lost at each of the postoperative time points. Continuous efficacy parameters and age in years were expressed as arithmetic mean ± standard deviation (SD), median, minimum, maximum, and 95% confidence interval. Comparisons between the postoperative time points and baseline, as well between the SE and FE when appropriate, were analyzed by paired *t*-test. Statistical significance was set at *p* < 0.05. Categorical demographic and categorical visual acuity parameters were summarized by absolute value and percentage. All data were analyzed using SAS v. 9.4 (SAS Institute, Cary, NC, USA). 

## 3. Results

### 3.1. Patient Baseline Demographics and Clinical Characteristics

In total, 24 patients participated in this study. Baseline demographics and pertinent clinical characteristics are outlined in Table 1. The study population comprised 13 (54.2%) females and 11 (45.8%) males. The mean age of the study population at enrollment was 77.0 ± 6.16 years (median: 76.0 years, range: 69.0–91.0 years). All patients were Caucasian. The majority of patients presented with geographic atrophy in the study eye. All measurable outcome parameters, i.e., CDVA, CDNVA, ECD, IOP, and ACD, were comparable between the study and fellow eyes at baseline.

### 3.2. Anterior Chamber Depth and Intraocular Pressure

**Figure 1:**
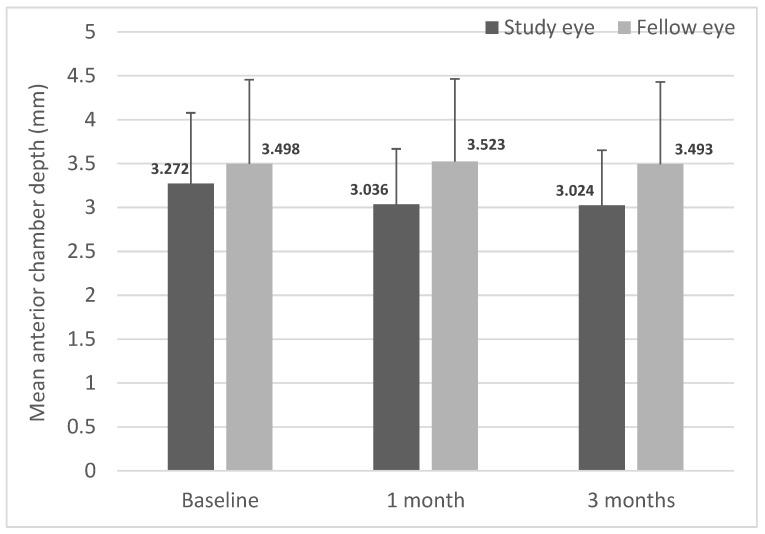
Mean anterior chamber depth (ACD) in study and fellow eyes over 3 months. Anterior chamber depth was measured preoperatively (baseline) and at months 1 and 3 postoperatively (*n* = 24). Mean anterior chamber depth was significantly reduced in the study eyes compared to the fellow eyes at 1 and 3 months post-SING IMT™ implantation. Data are expressed as mean ± standard deviation.

Mean ACD was comparable between the study and fellow eyes at baseline (3.272 ± 0.8063 mm and 3.498 ± 0.9577 mm, respectively; Figure 1). By postoperative months 1 and 3, the mean ACDs of the study eyes were 3.036 ± 0.6317 mm and 3.024 ± 0.6277 mm, respectively, which were not significantly different from baseline. Similarly, the mean ACD of the unoperated fellow eyes also remained unchanged from baseline.

**Figure 2:**
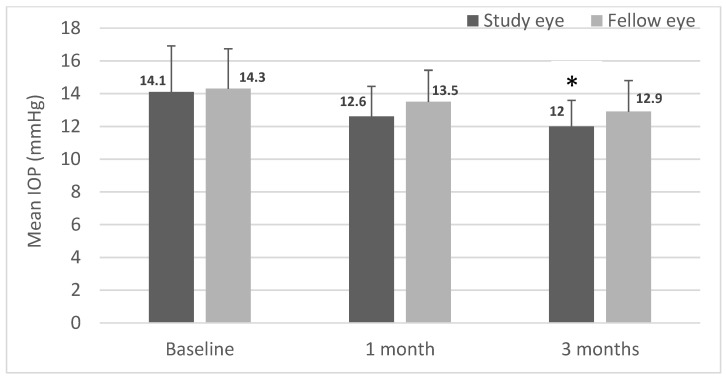
Mean intraocular pressure (IOP) in study and fellow eyes over 3 months. Intraocular pressure (IOP) was measured by Goldmann applanation tonometry preoperatively (baseline) and at months 1 and 3 postoperatively (*n* = 24). Data are expressed as mean ± standard deviation (* *p* < 0.05 from baseline; paired *t*-test).

While there was a significant decrease in IOP in the study eye from baseline at the 3-month time point, IOP was not significantly different between eyes at any of the time points (Figure 2). These data show that SING IMT™ implantation per se seems to have a significant impact on IOP, at least in the short term.

### 3.3. Visual Acuity Outcomes

At baseline, both the study and fellow eyes had comparable CDVA (Table 1). By months 1 and 3, the study eye had significantly higher CDVA than the fellow eye (*p* < 0.0001 for both). The average change in study eye CDVA from baseline was +7.3 ± 5.1 letters (*p* < 0.0001) at 1 month, increasing to +14.9 ± 7.1 letters (*p* < 0.0001) at 3 months. In comparison, the fellow eye experienced no appreciable CDVA change from baseline, with −0.1 ± 1.1 letters and 0.0 ± 0.8 letters at 1 and 3 months, respectively. 

Overall, by postoperative month 3, 20.8% and 54.2% of fellow eyes either experienced a loss in CDVA (defined as any loss in CDVA from baseline) or saw no change in CDVA from baseline, respectively. Only 25.0% of fellow eyes saw an improvement in CDVA, with all gaining fewer than five letters. Conversely, all study eyes demonstrated an improvement in CDVA from baseline by month 3, with 70.8% of eyes gaining 10 letters or more, 58.3% of eyes gaining 15 letters or more, 25.0% of eyes gaining 20 letters or more, and 8.3% of eyes gaining at least 30 letters (Table 2). 

Baseline mean CDNVA was also comparable between study and fellow eyes (Table 1). Mean CDNVA in the study eyes increased by 4.0 ± 2.65 J-levels (*p* < 0.0001) and 7.7 ± 3.17 J-levels (*p* < 0.0001) at 1 and 3 months, respectively. Indeed, 100% and 33% of study eyes saw an improvement in CDNVA ≥ 2 and ≥ 10 J-levels, respectively (Table 2).

### 3.4. Endothelial Cell Density

**Figure 3:**
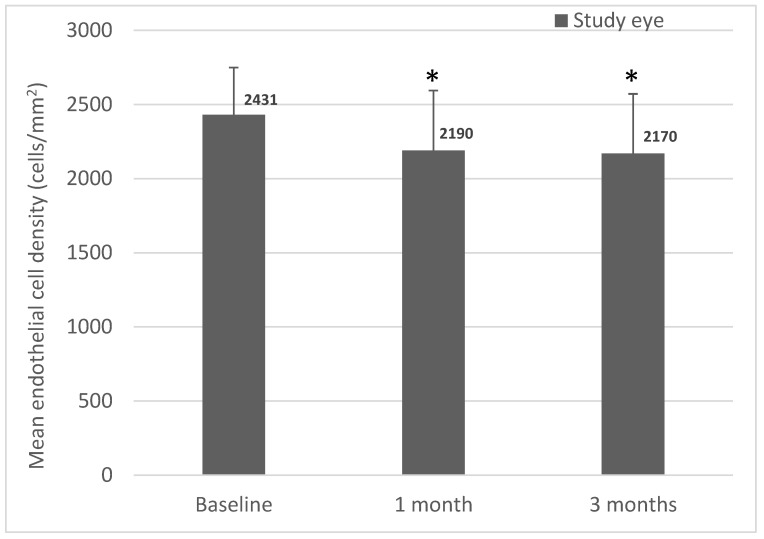
Mean endothelial cell density in study eyes over 3 months. Endothelial cell density was measured preoperatively (baseline) and at postoperative months 1 and 3 (*n* = 24). Data are expressed as mean ± standard deviation (* *p* < 0.05 from baseline, paired *t*-test).

Mean ECD was comparable between the study and fellow eyes at baseline. Compared to baseline, significant ECD loss occurred over the 3-month study in the study eyes. In the study eyes, there was an average ECD loss of 9.6 ± 13.3% at 1 month (*p* = 0.0047) and 10.4 ± 13.3% at 3 months (*p* = 0.0025) (Figure 3). 

### 3.5. Safety Outcomes

Commonly reported complications and adverse events (AEs) reported during the study are summarized in Table 3. Postoperative corneal edema was observed in over one-quarter of cases within the first month after surgery, however, this was resolved through a regimen of topical steroids and hypertonic eyedrops. There was only one case of corneal edema in the superior quadrant and surrounding the suture area persisting for longer than 30 days after surgery. This was resolved with standard pharmacological treatment by postoperative month 2. Cases of iris incarceration, atrophy, damage, and prolapse comprised most of the other complications, but were relatively uncommon. Iris incarceration and prolapse were treated with surgery. The two cases of iris atrophy were diagnosed within 7 days of surgery. 

A total of 14 ocular AEs were reported in seven patients (29.17%), and almost 70.00% of patients did not report any AEs. The most common AEs were inflammatory deposits on the device and distorted pupils, both reported in three patients (12.50%). Reactivation of CNV and iritis were reported in two patients (8.33%) and one patient (4.17%), respectively. Most AEs were considered possibly or probably related to the device, whereas CNV and increased IOP were considered unlikely to be related to the device. All AEs reported were expected of this type of implant, and most resolved with medical or surgical treatments. There were no reports of retinal detachment or endophthalmitis.

There were no aborted surgeries or conversions, and no device malfunctions were reported during the study period. 

## 4. Discussion

This study evaluated the visual outcomes and safety of the SING IMT™ telescope prosthesis, the second generation of the IMT™ implant, in patients with bilateral moderate to profound central vision impairment due to advanced AMD. To our knowledge, it is the first and largest single-surgeon case series in Europe to report short-term outcomes with the SING IMT™ device. These patients will continue to be monitored up to 12 months post-implantation. Notably, improvements to the device resulted in considerably lower ECD loss while maintaining the visual acuity outcomes achieved with the first-generation IMT™ device [14]. 

A defining advancement of the SING IMT™ implant is the potential for increased safety of the device and ease of use compared to its predecessor. The preloaded device generated a smaller corneal wound (7.5–8 mm vs. 10–12 mm), which could make the procedure less invasive, safer, and improve postoperative recovery. Indeed, compared to the cumulative incidence of increased IOP in the IMT-002 pivotal trial (28.0%), the rate in our study (4.2%) was notably lower [14]. Moreover, mean postoperative IOP remained comparable in both the study and fellow eyes in this study, suggesting no unintended induction of glaucomatous changes, further reinforcing the safety of the SING IMT™. 

We also showed lower rates of inflammatory and pigment deposits in our study compared to the pivotal trial; however, the rate of postoperative corneal edema was higher. A few potential reasons for this could include a more advanced cataract (the retrospective nature of his study meant that these data were not collected), a study cohort that was smaller and skewed older compared to that of the pivotal trial (range = 55–93 years), and the learning curve of the surgeon with the SING IMT™. While the rate of postoperative corneal edema is concerning, all cases but one were resolved with topical treatments over 2 months. This edema was also not indicative of ECD loss, which was considerably lower in our study than previously reported for the IMT™ (discussed in detail below) [14]. We noted two cases of CNV reactivation and one case of iritis persisting beyond the first postoperative month, however, these are somewhat expected complications, particularly given the inherent risk of intraocular inflammation with these procedures. Moreover, there is some evidence that the risk of incident CNV increases following cataract surgery [18,19,20,21,22,23]. A sizeable number of AEs and complications were associated with the iris, likely due to the degree of iris manipulation that was needed to correctly position the device. Indeed, iatrogenic iris defects are also a common complication of cataract surgery [24]. Techniques to prevent and minimize this damage with the SING IMT™ will improve with additional experience with implanting the device.

A key parameter relating to safety outcomes of any intraocular surgery is the rate of iatrogenic ECD loss, as this can lead to postoperative corneal decompensation, which in some cases could require addition surgical intervention, e.g., keratoplasty. With the IMT™, mean ECD loss was 20% at 3 months and 25% at 1 year [14]. Although this stabilized within 2 years, resulting in an annualized rate of 3% over 5 years, long-term ECD loss ranged between 35–40%, with younger patients (65–75 years) experiencing a lower rate of ECD loss compared to those aged 75 years and above [16]. Comparatively, we have been able to achieve much lower rates of ECD loss in this study with the SING IMT™, i.e., 10.4% at 3 months, effectively halving the loss. This is likely due to the improvements in the design of the SING IMT™, i.e., a smaller corneal wound, a foldable implant, a reduced need for intraocular device manipulation, and shorter operating times. While it remains to be seen whether additional ECD loss occurs over longer time periods, it is expected that ECD loss will stabilize following this initial loss. Importantly, this rate of ECD loss is comparable to that seen in modern cataract surgery with IOL implants [25]. Indeed, eyes that have undergone cataract surgery are known to lose endothelial cells at an annual rate of 2.5%, which is four times greater than the physiological rate of 0.6% per year observed in healthy unoperated eyes [26,27]. Overall, in this case series, the SING IMT™ has demonstrated equivalent if not better safety outcomes compared to the IMT™.

All of the patients in this study demonstrated improved CDVA and CDNVA in the study eye between their baseline and 3-month follow-up visits. Statistically significant improvements from baseline in mean BCVDA and CDNVA were observed as early as postoperative month 1. The mean BCVDA gain of +14.9 ± 7.1 letters (approximately 3 lines) in the study eyes over 3 months was similar to the mean 3.47-line improvement observed at 12 months in the IMT™ pivotal study [14]. Similarly, the IMT-002 pivotal study evaluating the IMT™ reported 66.7% gaining three or more lines (doubling of the visual angle) in 12 months, which is only slightly higher than the 58.3% of eyes that achieved the same degree of CDVA improvement in our study, but within 3 months. The mean CDNVA gain in the pivotal study was 3.18 lines at 12 months, although we observed a 7.7 J-level gain in our patients at 3 months. We expect that with additional visual rehabilitation sessions, CDNVA gains will improve. Indeed, the patients in our study had only received four sessions before CDNVA was assessed, whereas those in the pivotal trial participated in six sessions to occur in the longer term [14]. In all, short-term visual outcomes with the SING IMT™ were clinically significant, and so far already appear to be in line with those observed with the IMT™ at 12 months. 

This study is limited by its retrospective design and small patient cohort, the latter of which is an inevitable result of a relatively narrow indication for the device. However, a key strength of the study is the minimization of bias and variability resulting from a single surgeon performing all device implantations. 

In conclusion, implantation of the SING IMT™ telescope prosthesis in patients with bilateral advanced AMD demonstrated stability of a key indicator of corneal health, i.e., ECD loss comparable to modern cataract extraction procedures, and seems not to raise any unexpected safety signals. In addition, both distance and near visual acuity were significantly improved in the study eyes, suggesting that the SING IMT™ is at least as effective as its predecessor, the IMT™, in the short term. Taken together, the SING IMT™ telescope prosthesis appears to be safe and effective when used as intended and represents a significant advancement in the low vision rehabilitation for patients suffering from moderate to profound vision loss due to advanced AMD. 

## Figures and Tables

**Table 1:** Baseline demographics and clinical characteristics of the study population.

Characteristic	Value
Number of successfully implanted patients	24
Age (years)	
Mean (SD)	77.0 (6.2)
Range	69–91 years
Gender	
Female	13 (54.2%)
Male	11 (45.8%)
Study eye	
Right	16 (66.7%)
Left	8 (33.3%)
Race	
Caucasian	24 (100.0%)
Mean CDVA (ETDRS letters) ± SD	
Study eye	8.5 ± 7.3
Fellow eye	7.4 ± 7.2
Mean CDNVA (Jaeger levels) ± SD	
Study eye	15.6 ± 3.9
Fellow eye	15.2 ± 2.8
Mean ECD (cells/mm^2^) ± SD	
Study eye	2431.0 ± 318.5
Fellow eye	2309.6 ± 452.6
Mean IOP (mmHg) ± SD	
Study eye	14.1 ± 2.8
Fellow eye	14.3 ± 2.4
Mean ACD (mm) ± SD	
Study eye	3.272 ± 0.806
Fellow eye	3.498 ± 0.958
Macular lesion (study eye)	
Disciform scar associated with CNV	5 (20.8%)
Geographic atrophy	19 (79.2%)

SD = standard deviation; CDVA = best-corrected distance visual acuity; CDNVA = best-corrected near visual acuity; ECD = endothelial cell density; IOP = intraocular pressure; ACD = anterior chamber depth; CNV = choroidal neovascularization. Unless otherwise indicated, data are expressed as number (percentage) of participants.

**Table 2:** Cumulative proportion of study eyes that experienced a change in CDVA (ETDRS lines) and CDNVA (Jaeger levels) from baseline at 3 months after SING IMT™ implantation.

Change at 3 Months.	CDVA	CDNVA
	100.0% (+ 1 line)	100.0% (≥ 2 J-levels)
	70.8% (+ 2 lines)	87.5% (≥ 4 J-levels)
	58.3% (+ 3 lines)	79.2% (≥ 6 J-levels)
	25.0% (+ 4 lines)	41.7% (≥ 8 J-levels)
	8.3% (+ 5 lines)	33.3% (≥ 10 J-levels)

CDVA = best-corrected distance visual acuity; CDNVA = best-corrected near visual acuity; J = Jaeger.

**Table 3:** Ocular complications and adverse events in study and fellow eyes and the respective treatments administered.

Event	Number	Treatment Administered
Ocular complications		
Corneal edema within 30 days	7	Topical steroid and hypertonic eyedrops
Transient hyphema (within 30 days)	2 (intraoperative)	Anterior chamber BSS washing
	1 (postoperative)	-
Iris incarceration	3	Surgical repositioning
Iris atrophy within 7 days	2	-
Increasing IOP within 7 days, requiring treatment	1	Brinzolamide 1% and timolol 0.5% eyedrops
Iris damage	1	-
Iris prolapse	1	Surgical repositioning
Ocular adverse events		
Inflammatory deposits on device	3	Topical steroid eyedrops
Distorted pupil	3	Iridoplasty during suture removal
CNV	2	Intravitreal anti-VEGF injections
Iris atrophy > 7 days after surgery	2	-
Pigment deposits on device	2	-
Corneal edema > 30 days after surgery	1	Topical steroid and hypertonic eyedrops
Iritis > 30 days after surgery	1	Topical steroid eyedrops

CNV = choroidal neovascularization; VEGF = vascular endothelial growth factor; IOP = intraocular pressure; BSS = balanced salt solution. Unless otherwise indicated, data are expressed as number (percentage) of participants.

## Data Availability

Data are available upon reasonable request to the corresponding author.
